# Editorial: Women's health in an interdisciplinary dimension – determinants of nutritional disorders: Volume II

**DOI:** 10.3389/fnut.2026.1844748

**Published:** 2026-04-30

**Authors:** Karolina Krupa-Kotara, Patxi León-Guereño, Mateusz Rozmiarek

**Affiliations:** 1 Department of Epidemiology, Faculty of Public Health in Bytom, Medical University of Silesia, Katowice, Poland; 2 Universidad de Deusto, Bilbao, Spain; 3 Department of Sports Tourism, Faculty of Physical Culture Sciences, Poznan University of Physical Education, Poznań, Poland

**Keywords:** lifestyle factors, menopause, metabolic health, microbiome, nutritional disorders, nutritional epidemiology, preventive nutrition, reproductive health

## 1 Introduction

Women's health is among the most rapidly evolving areas of contemporary scientific research, particularly given the increasing prevalence of diet-related diseases and the complex interactions among biological, environmental, and sociocultural factors. The term “nutritional disorders” as used throughout this Research Topic refers broadly to diet-related metabolic conditions, micronutrient imbalances, and nutrition-associated health outcomes across the life course, rather than exclusively to clinically defined eating disorders. The extension of women's life expectancy has shifted lifestyle patterns, and global demographic transformations have redirected research priorities from single-disease entities toward systemic approaches that encompass the full spectrum of health determinants. These nutrition-related and metabolic conditions, including obesity, autoimmune diseases, reproductive challenges, and menopause-related changes, are increasingly analyzed within a multifactorial etiological framework in which both molecular mechanisms and psychosocial factors play central roles. Recent global analyses of non-communicable diseases, together with growing insight into sex-specific health trajectories, indicate across the life course, reinforcing the need for integrated epidemiological and clinical frameworks.

Modern medicine and nutritional science emphasize that health differences between women and men arise not only from biological sex but also from social and cultural determinants. Social roles, economic inequalities, access to healthcare, and lifestyle patterns shape health behaviors and may modify disease risk across every stage of a woman's life, from the reproductive period, through pregnancy and menopause, to aging. Concurrent advances in areas such as microbiome research, metabolic science, and selected nutrigenomic perspectives indicate that the nutritional environment can modulate gene expression and disease trajectories, reinforcing the need for an interdisciplinary research approach. In this context, interdisciplinarity refers not only to thematic diversity but also to the integration of methodological perspectives, including nutritional epidemiology, clinical endocrinology, behavioral sciences, and population health analytics.

The Research Topic “*Women's Health in an Interdisciplinary Dimension – Determinants of Nutritional Disorders: Volume II*” was developed in response to the growing demand for a platform that integrates diverse research perspectives within women's health. The articles collected in this volume cover a broad range of themes, including social determinants and lifestyle factors; reproductive health and metabolic disorders; autoimmune diseases; nutritional epidemiology; and the psychological aspects of aging. The methodological diversity of contributions, encompassing epidemiological analyses, cohort studies, and narrative reviews, reflects the evolution of contemporary women's health research toward the integration of clinical sciences, public health, and social sciences. Across the included studies, a recurring conceptual axis emerges linking adiposity, metabolic regulation, oxidative stress, and psychosocial determinants, illustrating how biological and social exposures converge in shaping women's nutritional health.

The aim of this Research Topic is not only to present current research findings but also to identify emerging directions in the prevention, diagnosis, and treatment of nutritional disorders in women. A unifying element across all publications is the emphasis on a holistic framework that integrates biological disease mechanisms with environmental and psychosocial contexts, enabling more precise design of health interventions and preventive strategies. Vol. II includes 10 articles that collectively present a broad interdisciplinary perspective on women's health. These studies can be organized into several key thematic areas, illustrating both biological and socio-environmental determinants of nutritional disorders.

## 2 Social determinants of health and inequalities in access to healthcare

The study by Roy et al. focuses on social barriers and perceptions of health services among women of reproductive age in India. Using a qualitative approach, the authors identified complex factors that hinder adherence to anemia-prevention strategies, including limited health literacy, limited access to iron supplementation, heavy domestic workloads, and cultural norms influencing dietary choices. This article contributes to the discussion of the social determinants of women's health by demonstrating that the effectiveness of nutritional interventions depends not only on medical recommendations but also on social context and healthcare system infrastructure.

## 3 Reproductive health and metabolic determinants of fertility

In reproductive health, Yu et al. examine the association between the Body Roundness Index and reproductive outcomes in women with polycystic ovary syndrome. The authors report that visceral adiposity was associated with reproductive outcomes and may represent a potentially informative anthropometric marker in the context of infertility treatment. Huang et al. propose a population-based approach by analyzing the cumulative effect of lifestyle factors on infertility risk. Their findings suggest that the combined assessment of diet, physical activity, sleep, and anthropometric parameters provides a more comprehensive understanding of biological mechanisms than analyses based on single risk factors. Both studies underscore the growing importance of metabolic prevention strategies in reproductive medicine.

## 4 Thyroid diseases and the role of antioxidant vitamins in autoimmune processes

Liang et al. examine the status of vitamins A and E in women with Hashimoto's thyroiditis, with particular attention to their associations with thyroid function parameters and markers of autoimmune activity. The authors report that alterations in antioxidant balance may be linked to the pathophysiological processes observed in autoimmune thyroid disease; however, these findings should be interpreted as indicative of potential relationships rather than direct clinical implications. The assessment of fat-soluble vitamin levels is therefore presented as a basis for further investigation rather than a diagnostic application. This contribution broadens the discussion of women's nutrition by situating micronutrient status within an immunoendocrinological context.

## 5 Nutritional deficiencies and their neurological and cardiovascular consequences

Hung et al. conducted a population-based cohort analysis indicating that iron-deficiency anemia was associated with a higher incidence of tinnitus in women. Their findings suggest that micronutrient insufficiency may be linked to auditory outcomes through proposed mechanisms, including reduced oxygen delivery, altered cochlear microcirculation, and systemic hemodynamic changes, but remain interpretive rather than mechanistically conclusive. In a complementary epidemiological context, Luo et al. evaluated the global burden of ischemic stroke attributable to inadequate intake of polyunsaturated fatty acids in older women. Their projections highlight the contribution of suboptimal dietary fat composition to cerebrovascular risk and highlight the potential preventive relevance of adequate PUFA consumption within cardiovascular health strategies.

## 6 Nutritional epidemiology and artificial intelligence-based analytical approaches

Zhang et al. employed population-based data and machine learning models to examine the relationship between the dietary antioxidant index and HPV infection. The study reports an association between higher antioxidant intake and reduced infection risk in predictive modeling frameworks, while the analytical approaches employed identify key risk determinants in large populations without implying causal relationships. This work illustrates the growing role of artificial intelligence–driven analytics in advancing data-oriented research in women's nutritional health.

## 7 Eating behaviors and obesity phenotypes in young women

Walkiewicz et al. present a narrative review exploring eating behavior patterns in relation to obesity phenotypes and beige adipose tissue content in young women. The authors discuss potential metabolic mechanisms and the role of diet quality in regulating thermogenesis and energy metabolism. The article provides an important perspective on early obesity prevention and highlights the role of lifestyle factors in modulating body composition.

## 8 Integrative therapeutic strategies in midlife women's health

Dahia et al. review the use of natural health products (NHPs) as complementary therapies among midlife women from a multidisciplinary perspective. The authors emphasize the need for standardized, well-designed clinical research frameworks to evaluate efficacy, safety, bioavailability, and the activity spectrum of different extracts, and the importance of integrating NHPs into evidence-based clinical management. The paper also highlights the role of effective communication of scientific evidence, health professional education, and interdisciplinary collaboration in response to the growing interest in natural therapeutic approaches.

## 9 Mental and metabolic health in postmenopausal women

Krupa-Kotara et al. analyze the relationship between metabolic health indicators and self-esteem among postmenopausal women. The findings reveal that unfavorable anthropometric and metabolic parameters are associated with lower psychological wellbeing, highlighting the importance of a biopsychosocial approach in the care of aging female populations. The authors also emphasize the role of physical activity in supporting mental health.

While the studies included in this Research Topic provide valuable perspectives across multiple domains of women's health, several methodological considerations warrant acknowledgment. The collected evidence reflects heterogeneous study designs, with a substantial proportion based on observational or exploratory analytical frameworks. In addition, geographic representation remains uneven, which may influence the generalizability of findings across diverse populations. These aspects should be considered when interpreting the broader clinical and public health implications discussed in this volume.

## 10 Summary

The articles included in this volume confirm that women's health must be approached from a multidimensional perspective that integrates biological, environmental, social, and behavioral determinants. The nutritional disorders discussed in this volume, including a range of diet-related diseases, do not arise from single risk factors but rather from dynamic interactions among lifestyle patterns, metabolic status, psychosocial conditions, and access to healthcare. A central conclusion from these studies is the need to move beyond reactive treatment models toward preventive strategies grounded in early risk identification and multilevel interventions.

Research findings in reproductive and metabolic health suggest a growing relevance of precise anthropometric indicators and comprehensive lifestyle assessment in the clinical management of women of reproductive age. Integrating metabolic evaluation with behavioral analysis may facilitate personalized dietary recommendations and improve outcomes in infertility treatment. Simultaneously, studies on autoimmune conditions and micronutrient deficiencies emphasize the importance of routine nutritional status assessment in clinical practice, particularly in chronic diseases and endocrine disorders.

Another key implication concerns diet quality, including antioxidant intake and polyunsaturated fatty acids, in the prevention of chronic diseases and viral infections. The integration of artificial intelligence-based analytical tools into population research offers new opportunities for identifying high-risk groups and designing more targeted preventive programs. At the same time, studies on social determinants highlight that the effectiveness of nutritional interventions is strongly influenced by cultural and structural contexts, underscoring the need for collaboration among public health, education, and social policy sectors.

From a clinical and public health perspective, the collected works indicate several priority directions. First, lifestyle assessment and psychosocial evaluation may represent valuable components of future diagnostic frameworks in women's health. Second, the development of integrative care models that combine nutrition science, lifestyle medicine, and psychosocial interventions may improve health outcomes across life stages. Third, research on older women emphasizes the importance of designing programs that promote physical activity and healthy aging while addressing both somatic health and psychological wellbeing.

In conclusion, Vol. II highlights the importance of interdisciplinary approaches in women's health research and demonstrates the need to integrate knowledge from nutritional epidemiology, endocrinology, public health, and social sciences. Translating the findings presented in this Research Topic into clinical practice and public health strategies may contribute to more effective prevention of nutritional disorders, reduction of health inequalities, and improvement in women's quality of life worldwide.

